# Enhancing the structure–function relationship in glaucoma using anatomical compensation of retinal nerve fibre layer

**DOI:** 10.1136/bjo-2023-324792

**Published:** 2024-05-07

**Authors:** Jacqueline Chua, Chi Li, Rachel Chong, Tina T Wong, Rahat Husain, Tin Aung, Leopold Schmetterer, Damon Wing Kee Wong

**Affiliations:** 1 Singapore Eye Research Institute, Singapore National Eye Centre, Singapore; 2 NTU-SERI, Singapore; 3 Glaucoma, Singapore National Eye Centre, Singapore; 4 Singapore National Eye Centre, Singapore; 5 SERI-NTU Advanced Ocular Engineering (STANCE), Singapore; 6 School of Chemistry, Chemical Engineering and Biotechnology, Nanyang Technological University, Singapore; 7 Ocular Imaging, Singapore Eye Research Institute, Singapore; 8 Singapore Eye Research Institute, Singapore

**Keywords:** Glaucoma, Retina, Imaging

## Abstract

**Background/aims:**

To investigate whether compensating retinal nerve fibre layer (RNFL) thickness measurements for demographic and anatomical ocular factors can strengthen the structure–function relationship in patients with glaucoma.

**Methods:**

600 eyes from 412 patients with glaucoma (mean deviation of the visual field (MD VF) −6.53±5.55 dB) were included in this cross-sectional study. Participants underwent standard automated perimetry and spectral-domain optical coherence tomography imaging (Cirrus; Carl Zeiss Meditec). Compensated RNFL thickness was computed considering age, refractive error, optic disc parameters and retinal vessel density. The relationship between MD VF and RNFL thickness measurements, with or without demographic and anatomical compensation, was evaluated sectorally and focally.

**Results:**

The superior arcuate sector exhibited the highest correlation between measured RNFL and MD VF, with a correlation of 0.49 (95% CI 0.37 to 0.59). Applying the compensated RNFL data increased the correlation substantially to 0.62 (95% CI 0.52 to 0.70; p<0.001). Only 61% of the VF locations showed a significant relationship (Spearman’s correlation of at least 0.30) between structural and functional aspects using measured RNFL data, and this increased to 78% with compensated RNFL measurements. In the 10°–20° VF region, the slope below the breakpoint for compensated RNFL thickness demonstrated a more robust correlation (slope=1.66±0.18 µm/dB; p<0.001) than measured RNFL (slope=0.27±0.67 µm/dB; p=0.688).

**Conclusion:**

Compensated RNFL data improve the correlation between RNFL measurements and VF parameters. This indicates that creating structure-to-function maps that consider anatomical variances may aid in identifying localised structural and functional loss in glaucoma.

**WHAT IS ALREADY KNOWN ON THIS TOPIC**

- Prior to this study, it was recognised that retinal nerve fibre layer (RNFL) thickness measurements were crucial for assessing glaucoma. However, existing research did not comprehensively consider demographic and anatomical factors when studying the structure–function relationship in glaucoma.

**WHAT THIS STUDY ADDS**

- This study introduces a novel approach of compensating RNFL measurements for demographic and anatomical ocular factors, significantly enhancing the correlation with visual field loss in patients with glaucoma. It reveals that such compensation improves the detection of structural and functional impairment. The findings shed light on a more accurate and effective method for assessing glaucoma.

**HOW THIS STUDY MIGHT AFFECT RESEARCH, PRACTICE OR POLICY**

- The results suggest that adopting compensated RNFL data in clinical practice can lead to more precise glaucoma diagnosis and monitoring. It emphasises the importance of considering anatomical variances when evaluating structure–function relationships in glaucoma. This study may influence future research directions and potentially guide policy changes for glaucoma management.

## Introduction

 The relationship between structural and functional changes in the eye is important for glaucoma diagnosis and management.^1^ In glaucoma, the progressive loss of retinal ganglion cells and their axons leads to thinning of the peripapillary retinal nerve fibre layer (RNFL), which can be detected using optical coherence tomography (OCT).^2^ Despite being the gold standard for functional assessment, visual field (VF) loss is not well correlated with RNFL thickness, particularly in the early stages of glaucoma.^3^

Previously reported structure–function maps have provided insights into the relationship between optic nerve head (ONH) structure and visual function.^4^,^6^ Using the nerve fibre paths from the participants in their respective studies, generalised maps were statistically derived to obtain representative mappings between the structural locations around the ONH with functional VF test points. However, these maps lacked consideration for intraindividual differences, which are crucial. Individual variations in ocular parameters, such as optic disc area, can notably impact the structure–function relationship.^7^ Relying solely on generalised information may overlook these individual differences, leading to inaccurate conclusions about the connection between RNFL thickness measurements and VF function, especially in individuals with high myopia or tilted optic discs.

We developed a novel approach using a multivariate linear regression model to adjust RNFL thickness for demographic factors and anatomical ocular parameters.^8 9^ Our findings indicate that compensated RNFL thickness is more effective than conventional OCT-derived RNFL thickness in detecting early-stage glaucoma.^10^ Adjusting RNFL thickness for demographic and anatomical factors enhances accuracy in reflecting the true RNFL thickness, potentially improving the precision of the structure–function relationship between RNFL thickness and VF metrics.

This study examines whether our compensation model improves the structure–function correlation in glaucoma beyond standard RNFL thickness measurements. Analysing 600 eyes from 412 patients with glaucoma, we hypothesise that incorporating the compensation model will enhance sectoral and focal structure–function relationships compared with unadjusted RNFL thickness measurements.

## Methods

The present study was a cross-sectional, prospective investigation involving participants with glaucoma recruited from a clinic-based study conducted at the Singapore National Eye Centre in Singapore.

Subjects with glaucoma were recruited from a genetic investigation of primary open-angle glaucoma.^11^ Glaucoma was defined by the loss of neuroretinal rim, vertical cup-to-disc ratio >0.7, intereye asymmetry >0.2 or glaucoma-related notching. Participants performed Humphrey VF 24-2 testing and needed an abnormal glaucoma hemifield test result, reliability thresholds ≤20% for fixation losses, false positives, false negatives and the absence of secondary causes of optic neuropathy. Glaucoma severity was classified as mild (mean deviation (MD) ≥−6.00 dB), moderate (−6.01 to −12.01 dB) and advanced (MD <−12.01 dB).^12^ Participants underwent demographic interviews, medical history assessments and auto-refraction-keratometry measurements. The spherical equivalent, expressing refractive error, was calculated by adding half of the negative cylinder value to the spherical value.

### Optical coherence tomography

Participants underwent spectral-domain OCT imaging (Carl Zeiss Meditec, Dublin, California, USA) using the 200×200 optic disc cube scan protocol after pupil dilation with tropicamide 1%.^9^ The OCT scans were reviewed by trained graders blinded to clinical characteristics. Eyes with poor-quality images (signal strength <6, excessive movement artefacts, inconsistent signal intensity across the scan or segmentation artefacts) were excluded from the analysis. If both eyes of a participant met the eligibility criteria, both eyes were included in the study.

### RNFL thickness compensation model

Due to the unavailability of macular images, the current compensation model relies solely on optic disc images.^10^ The compensation model employs a multivariable linear regression analysis, adjusting RNFL thickness measurements for six variables: age,^9 13^ spherical equivalent,^14^ optic disc parameters (area, orientation and ratio) and retinal vessel density^15 16^ (online supplemental materials).

### Structure–function modelling

The study employed structure–function modelling, using deviation values from individual field points derived from the total deviation plot on a logarithmic scale for VF data analysis.

We conducted a sectoral analysis using the Garway-Heath map that corresponds ONH regions with VF test points.^4^ The ONH sectors, split into 360°, include temporal (311°–40°), superior temporal (41°–80°), superior nasal (81°–120°), nasal (121°–230°), inferior nasal (231°–270°) and inferior temporal (271°–310°), with 0° as the temporal reference. The mean values of each sector were then calculated for measured and compensated RNFL thicknesses.

For the pointwise focal map, we applied Jansonius *et al*’s nerve fibre trajectory model,^6^ describing retinal nerve fibre trajectories originating from the optic disc and associating each VF test location with specific fibre trajectory ranges. Chinese population averages of disc–fovea distance (4.59 mm) and disc–fovea angles (−7.46°) were used to adjust nerve fibre trajectories,^9^ aligning them with the optic disc location in the 6×6 mm OCT image.^17^ Each VF test location was associated with a 95% CI range of nerve fibre trajectories, defining a wedge-shaped region of interest (ROI). This ROI represents the nerve fibre bundle related to the test point, enabling focal structure–function analysis of the optic nerve. Focal nerve layer thickness was calculated for each ROI, providing focal structural-measured RNFL thickness and compensated RNFL thickness mean values within defined circumpapillary measurements. Focal maps were stratified into three eccentricities: <10° (11 VF test locations), 10°–20° (18 VF test locations) and >20° (22 VF test locations).

### Statistical analyses

Primary outcomes focused on the relationships between VF loss and RNFL thickness measurements. Spearman’s correlation coefficient (R_s_) assessed the correlation strength, with 95% CIs calculated using bootstrapping.^18^ To adjust for intereye correlation, we used a resampling technique (bootstrap) with a custom-paired bootstrap resampling approach. This involved randomly selecting one eye while maintaining the pairing structure to account for the correlation between measurements from both eyes. Global correlations assessed VF MD and global RNFL thickness, while sectoral correlations were based on Garway-Heath sectors. Focal pointwise correlations used a nerve fibre trajectory tracing model.^6^ Structure–function relationships for focal VF loss and individual RNFL thickness were employed using the breakpoint model and the anti-log model to assess the floor effect.^19^ For the breakpoint model, we used segmented regression to determine breakpoints and slopes, where a floor-like effect was defined as a non-significant slope below the breakpoint.^20 21^ The Davies test was used to determine the significance of a change in slope in the relationship, with an estimate of the breakpoint location where the change occurs.^22^ Locally weighted scatterplot smoother curves visualised structure–function relationships. For the anti-log model, we adopted the approach proposed by Hood and Kardon,^19^ in which the logarithmic VF values in decibels were converted to the equivalent anti-log values by unlogging the decibels. Linear regressions were then performed on the anti-log values to assess their relationship with the RNFL thickness values. Floor effects were assessed by considering the y-intercept of the regression. Participants were classified into three groups based on RNFL thickness: normal (green category), suspect (yellow category) and abnormal (red category).^10^ Statistical significance was set at p<0.05, and R software V.4.0.4 was used.

## Results

Of the initial 749 eyes with OCT and VF, 26 were excluded for poor VF reliability, 39 for low-quality OCT and 110 for missing refractive error, resulting in 600 eyes with reliable VF and good-quality OCT. The study focused on 412 participants, representing 600 eyes with glaucoma—353 (59%) with mild, 156 (26%) with moderate and 91 (15%) with advanced glaucoma. Participants had a mean age of 67±9 years, with 63% being male. The average MD value was −6.53±5.55 dB, and the average RNFL thickness was 75.49±12.52 µm. After applying the compensation model, the average deviation of RNFL thickness from normality was −21.02±12.58 µm. The characteristics of the participants are presented in online supplemental table 1.

Table 1 displays Spearman’s correlation coefficient for the Garway-Heath ONH sectoral map using measured and compensated RNFL thicknesses. In all glaucoma eyes, the linear relationship between the variables was stronger with compensated RNFL thickness (R_s_=0.10–0.62) compared with measured RNFL thickness (R_s_=0.08–0.49), indicating an improved structure–function relationship. This trend held across all glaucoma stages: mild glaucoma (R_s_=0.10–0.43 vs 0.09–0.30), moderate glaucoma (R_s_=0.09–0.58 vs 0.08–0.52) and advanced glaucoma (R_s_=0.18–0.48 vs 0.07–0.32).

**Table 1:** Spearman’s correlations between VF mean deviation and RNFL thickness

Optic nerve head sectors	Measured RNFL	Compensated RNFL	P value
All glaucoma			
Global*	0.33 (0.20 to 0.46)	**0.46 (0.35to 0.57**)	**<0.001**
Superior peripheral	0.27 (0.14 to 0.38)	**0.35 (0.23to 0.46**)	**0.001**
Nasal	0.08 (−0.06 to 0.21)	0.10 (−0.04 to 0.23)	0.542
Superior arcuate	0.49 (0.37 to 0.59)	**0.62 (0.52to 0.70**)	**<0.001**
Central	0.19 (0.05 to 0.32)	0.25 (0.12 to 0.37)	0.060
Inferior arcuate	0.40 (0.28 to 0.51)	**0.51 (0.40to 0.61**)	**<0.001**
Inferior peripheral	0.24 (0.11 to 0.35)	**0.30 (0.17to 0.41**)	**0.005**
Mild glaucoma			
Global*	0.13 (−0.04 to 0.28)	**0.21 (0.6to 0.37**)	**0.004**
Superior peripheral	0.09 (−0.08 to 0.25)	0.17 (0.00 to 0.33)	0.073
Nasal	0.00 (−0.17 to 0.16)	0.00 (-0.18 to 0.16)	0.759
Superior arcuate	0.30 (0.13 to 0.44)	**0.43 (0.29to 0.55**)	**<0.001**
Central	0.04 (−0.13 to 0.21)	0.10 (−0.06 to 0.26)	0.114
Inferior arcuate	0.25 (0.08 to 0.40)	**0.33 (0.17to 0.47**)	**0.005**
Inferior peripheral	0.11 (−0.06 to 0.26)	0.11 (−0.06 to 0.28)	0.908
Moderate glaucoma			
Global*	0.08 (−0.15 to 0.31)	0.19 (−0.05 to 0.40)	0.184
Superior peripheral	0.14 (−0.09 to 0.36)	0.09 (−0.13 to 0.31)	0.995
Nasal	0.09 (−0.13 to 0.32)	0.09 (−0.16 to 0.32)	0.895
Superior arcuate	0.37 (0.16 to 0.57)	0.50 (0.31 to 0.67)	0.055
Central	0.25 (−0.02 to 0.47)	0.17 (−0.11 to 0.40)	0.159
Inferior arcuate	0.52 (0.32 to 0.67)	0.58 (0.39 to 0.71)	0.579
Inferior peripheral	0.25 (0.00 to 0.47)	0.27 (0.01 to 0.48)	0.657
Advanced glaucoma			
Global*	0.07 (−0.24 to 0.38)	0.18 (−0.19 to 0.46)	0.194
Superior peripheral	0.17 (−0.19 to 0.45)	0.28 (−0.06 to 0.57)	0.071
Nasal	−0.03 (−0.34 to 0.29)	−0.08 −0.37 to 0.24)	0.392
Superior arcuate	0.16 (−0.16 to 0.46)	**0.30 (−0.04to 0.57**)	**0.024**
Central	−0.06 (−0.38 to 0.28)	0.00 (−0.33 to 0.31)	0.923
Inferior arcuate	0.32 (−0.02 to 0.58)	0.48 (0.15 to 0.71)	0.115
Inferior peripheral	0.12 (−0.20 to 0.40)	**0.23 (−0.07to 0.50**)	0.100

Hittner 2003 test is used to compare two overlapping correlations from dependent groups.

Data in parentheses are two-sided 95% custom-paired bootstrap CI for the true Spearman’s correlation.

Values in bold indicate statistically significant results and indicate the higher correlation when comparing between measured and compensated RNFL.

* Global mean deviation.

RNFLretinal nerve fibre layerVFvisual field

**Figure 1:**
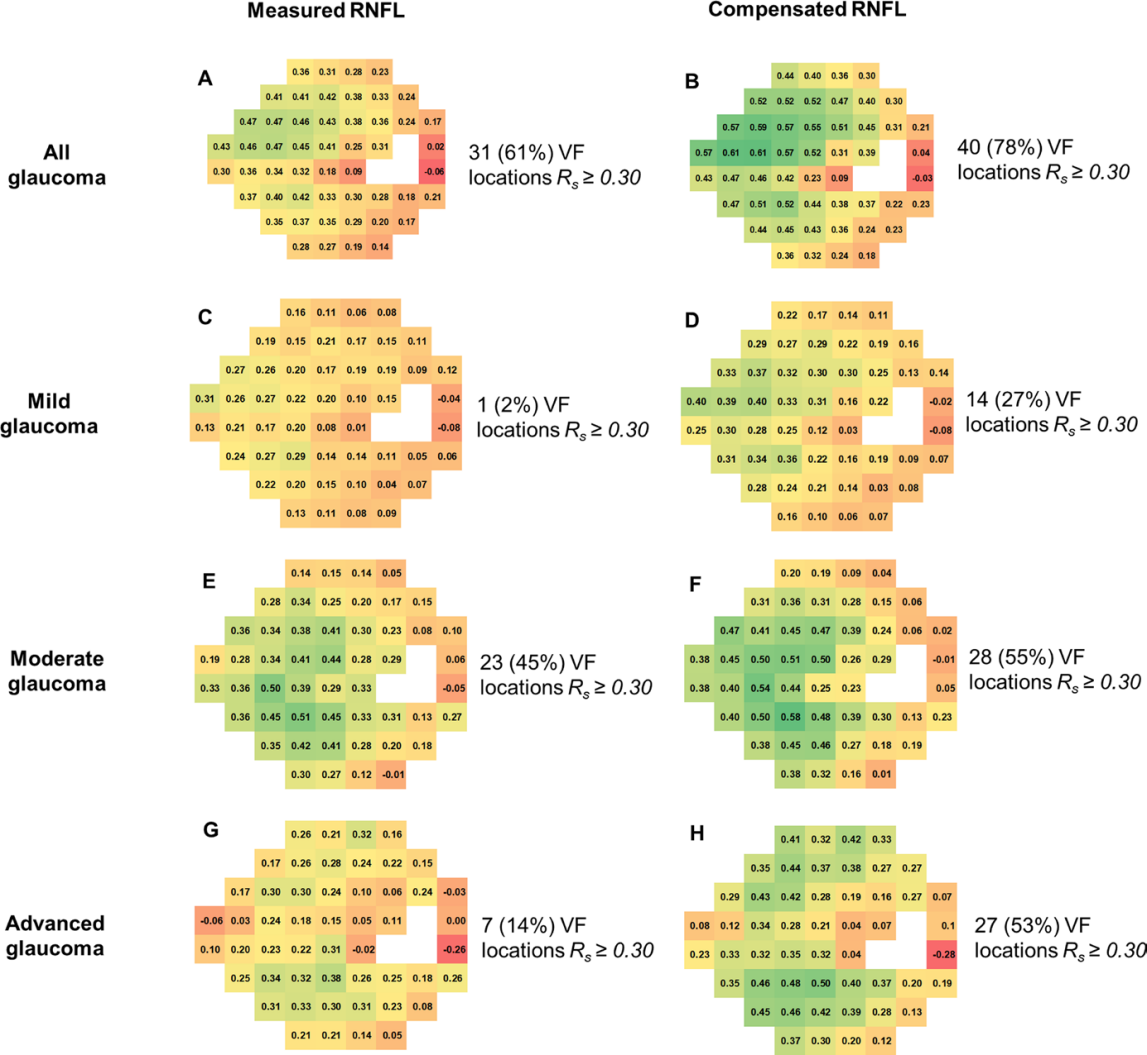
Focal structure–function models were used to analyse spatial distributions in detecting various stages of glaucoma using measured (A, C, E, G) and compensated (B, D, F, H) retinal nerve fibre layer (RNFL) thicknesses. The results showed that for all glaucoma cases, 40 (78%) visual field (VF) locations achieved at least a Spearman’s correlation value of R_s_ ≥0.30 with compensated RNFL, compared with only 31 VF locations (61%) with measured RNFL. Similar trends were observed in early glaucoma (27% vs 2%), moderate glaucoma (55% vs 45%) and advanced glaucoma (53% vs 14%). The compensated RNFL exhibited a stronger structure–function relationship than the measured RNFL. The maps indicate the right eye.

Figure 1 illustrates the spatial distributions of the models using focal maps. In all glaucoma eyes, 78% of VF locations showed an R_s_ of 0.30 or higher with compensated RNFL thickness (n=40), compared with 61% with measured RNFL thickness (n=31). This pattern persisted in early glaucoma (27% vs 2%), moderate glaucoma (55% vs 45%) and advanced glaucoma (53% vs 14%). Overall, compensated RNFL thickness exhibited a stronger structure–function relationship in more VF locations, particularly in the superior nasal field (corresponding to the temporal–inferior structural regions), compared with measured RNFL thickness.

Table 2 displays the focal VF measurements for both measured and compensated RNFL thicknesses in the different eccentricities. Statistical significance was observed for all identified breakpoints and for the slope above the breakpoint. Notably, the slope above the breakpoint was greater using compensated RNFL thickness (slope=2.31±0.75 µm/dB; p=0.004) compared with measured RNFL thickness (slope=1.64±0.76 µm/dB; p=0.030). This trend holds for other eccentricities as well, where compensated RNFL thickness (p<0.001 for all three regions) showed higher slope compared with measured RNFL thickness (p=0.014–0.020).

**Table 2:** Predicted breakpoints and corresponding slopes from segmented regression analysis

	VF breakpoint		Slope below breakpoint		Slope above breakpoint	
**Retinal eccentricity zone**	**Mean±SD**	P value*	**Mean±SD**	**P value**	**Mean±SD**	**P value**
All						
Measured RNFL	−16.90±2.88	**0.012**	−0.04±0.74	0.960	1.64±0.76	**0.030**
Compensated RNFL	−17.35±2.16	**<0.001**	−0.07±0.73	0.924	2.31±0.75	**0.004**
Less than 10°						
Measured RNFL	−13.18±3.03	**0.011**	0.34±2.88	0.469	1.29±0.53	**0.014**
Compensated RNFL	−13.27±1.62	**<0.001**	0.10±0.43	0.825	2.24±0.49	**<0.001**
Between 10° and 20°						
Measured RNFL	−16.00±3.01	**0.010**	0.27±0.67	0.688	1.64±0.70	**0.020**
Compensated RNFL	−3.61±0.74	**<0.001**	1.66±0.18	**<0.001**	3.37±1.01	**<0.001**
More than 20°						
Measured RNFL	−10.46±2.85	**0.017**	0.47±0.33	0.153	1.05±0.44	**0.018**
Compensated RNFL	−10.41±1.98	**<0.001**	0.85±0.37	**0.021**	1.68±0.49	**<0.001**

Values are expressed as mean±SD, unless otherwise indicated.

Values in bold indicate statistically significant results.

* Davies test for the existence of a breakpoint.

RNFLretinal nerve fibre layerVFvisual field

**Figure 2:**
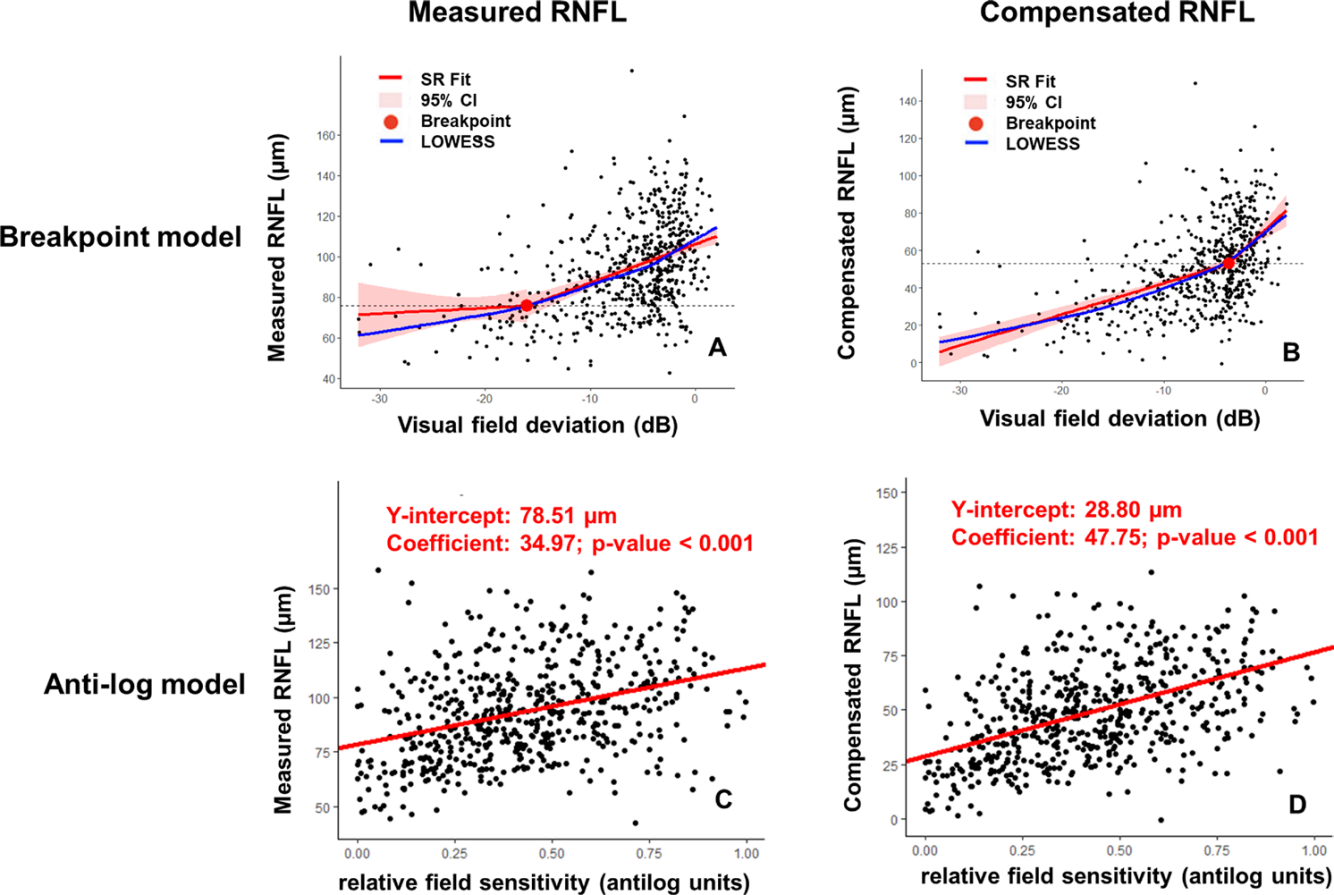
Scatterplots showing the association between visual field (VF) losses in retinal eccentricity between 10° and 20° using the breakpoint model (A,B) and anti-log Hood and Kardon model (C,D) when using measured (A and C) and compensated (B and D) retinal nerve fibre layer (RNFL) thicknesses. For the breakpoint model, locally weighted scatterplot smoothing (LOWESS, blue) curves are provided with fits obtained from segmented regression analysis (SR, red). For the anti-log model, linear mixed-effects model regression lines (adjusted for intereye correlation) were added to the scatterplot.

In the central 10° VF, no significant association was found between VF losses below the breakpoint and either compensated or measured RNFL thickness. However, in the 10°–20° region, the slope below the breakpoint for compensated RNFL thickness was significant (slope=1.66±0.18 µm/dB; p<0.001), while measured RNFL thickness was not (slope=0.27±0.67 µm/dB; p=0.688). Similarly, in the >20° region, compensated RNFL thickness was significant (slope=0.85±0.37 µm/dB; p=0.021), whereas measured RNFL thickness was not (slope=0.47±0.33 µm/dB; p=0.153). This suggests that compensated RNFL thickness overcomes the floor effect, particularly beyond 10° of VF, and may better predict focal VF loss in those regions than measured RNFL thickness. Scatterplots of focal VF against RNFL thickness within the 10°–20° regions using the breakpoint and the anti-log models are shown in figure 2. In the 10°–20° region, the coefficient was higher for compensated RNFL thickness (47.75, p<0.001) compared with measured RNFL thickness (34.97, p<0.001), and the y-intercept of the regression line for compensated RNFL thickness was lower (28.80 µm) compared with measured RNFL thickness (78.51 µm). Similarly, in the >20° region, the effect size was larger for compensated RNFL thickness (29.89, p<0.001) compared with measured RNFL thickness (22.12, p<0.001), and the y-intercept of the regression line for compensated RNFL thickness was lower (45.03 µm) compared with measured RNFL thickness (77.89 µm)

**Figure 3:**
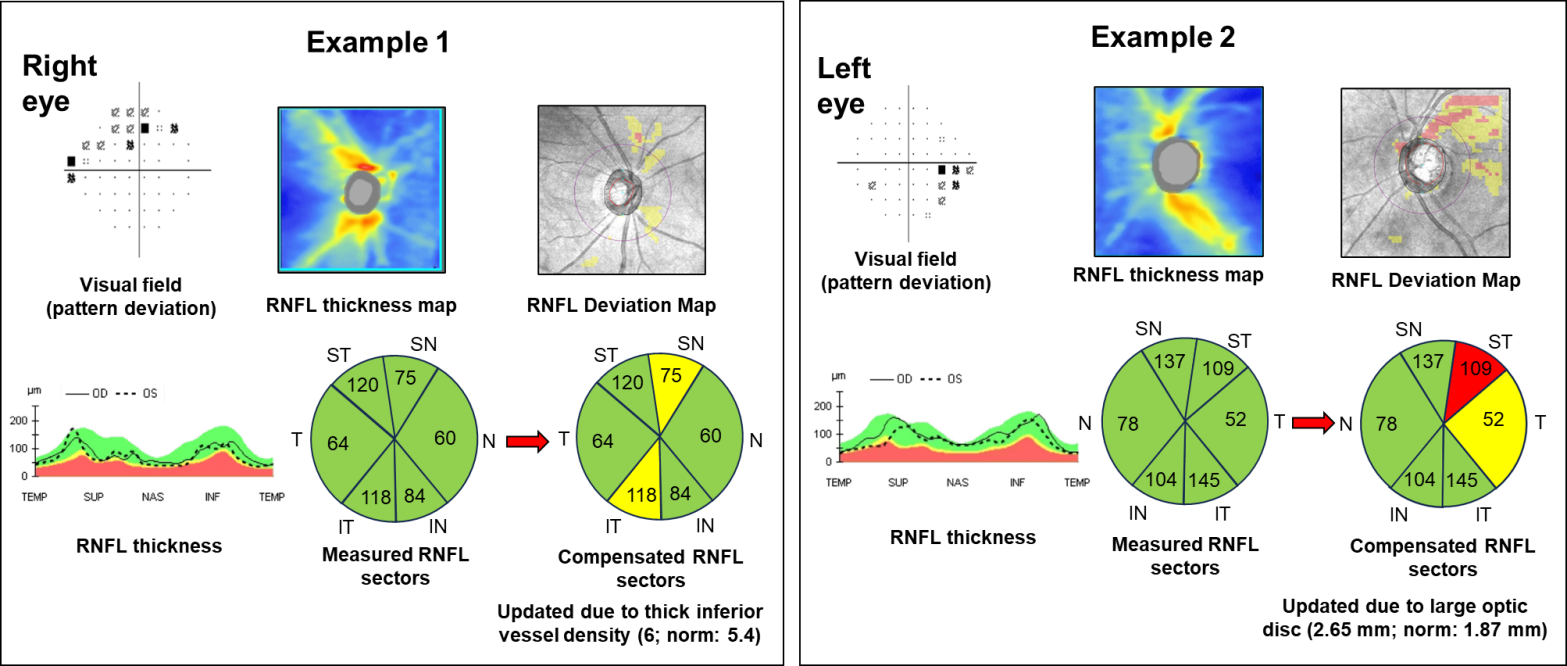
The study presented two cases of patients with glaucoma. The colour codes representing the retinal nerve fibre layer (RNFL) probability in the Garway-Heath six-sector map changed after applying the compensation model. In the first example, thicker inferior retinal vessels resulted in an overestimation of the RNFL thickness. In the second example, a large optic disc area led to an overestimation of the RNFL measurement. After compensating for these anatomical variations, the compensated RNFL measurements correlated better with the visual field abnormality. I, inferior; N, nasal; S, superior; T, temporal.

Figure 3 illustrates the improved correlation between VF and compensated RNFL thickness compared with measured RNFL thickness using the Garway-Heath ONH sectoral map. In both cases, the colour codes based on measured RNFL thickness were normal (green), but the compensation model resulted in changes. In the first example, the patient exhibited thicker than normal inferior retinal vessels, resulting in a higher RNFL thickness measurement than the normative database. After compensation, the inferotemporal region of the RNFL showed borderline thinning, which aligned with the superior arcuate VF defect. In the second example, the patient’s large optic disc area (2.65 mm) resulted in closer proximity to the OCT’s scan circle (3.46 mm), which led to a thicker RNFL measurement compared with the normative database (1.87 mm). After compensation, the superotemporal region was colour coded as red, showing a better correlation with the inferior nasal step.

## Discussion

In a cross-sectional study of 600 glaucoma eyes, applying an anatomically compensated RNFL thickness model significantly improved the structure–function relationship. Enhanced focal/sectoral analysis, particularly in superior and inferior nasal VF regions, and partial overcoming of the floor effect beyond 10° of VF were observed. These results indicate that compensated RNFL offers a more accurate representation of structural changes in glaucoma, improving our ability to correlate structural damage with functional impairment.

### Compensated RNFL thickness improves the structure–function relationship in glaucoma

The study highlights the efficacy of anatomically compensated RNFL thickness analysis in improving glaucoma assessment at various stages. In mild glaucoma, compensated RNFL thickness demonstrated a 25% increase in locations with correlation coefficients of 0.30 or higher compared with default RNFL thickness measurements. This compensation addresses individual anatomical variations, reducing the impact of confounding factors and enhancing the correlation between subtle structural changes and visual function. The analysis proves particularly valuable in early glaucoma detection, providing clinicians with more accurate insights.

One challenge in measuring RNFL thickness is the presence of non-neural elements like glial tissue and blood vessels, introducing variability.^6^ Compensated RNFL thickness analysis considers factors such as retinal vessel density, adjusting for vessel-related artefacts. This approach significantly expands the accuracy range, especially in advanced glaucoma, where structural measurements are crucial. The study emphasises the importance of anatomically compensated RNFL thickness analysis in advancing our understanding of how RNFL thickness changes affect visual function and improving diagnostic accuracy, particularly in advanced glaucoma.

### Compensated RNFL thickness mitigates the floor effects in glaucoma

Compensated RNFL thickness avoids the floor effect, especially beyond 10° of VF, which hinders the observation of further retinal tissue thinning in advanced glaucoma. The floor effect, associated with challenges in understanding the structure–function relationship in advanced cases, is partly complicated by blood vessels.^20 23^ Compensating for vessel-related artefacts helps overcome the floor effect, particularly beyond 10° eccentricities. While previous studies reported floors ranging from −10 dB to −14 dB,^17^ our analysis showed breakpoints but maintained a linear structure–function relationship with increasing VF loss beyond these points. Compensated RNFL thickness analysis, adjusting for anatomical variations, offers a more reliable assessment of neuronal tissue, improving the structure–function relationship beyond reported RNFL floors. However, despite its effectiveness, the compensated analysis could not eliminate floor effects within the central 10° retinal eccentricity, possibly due to limited data on advanced glaucoma cases in this region. Future research with a focus on this advanced subpopulation is needed.

In comparing the breakpoint model^24^ with the anti-log model,^19^ several limitations and advantages emerge. The breakpoint model assumes a change in the relationship between VF and RNFL data at a specific point (the breakpoint), allowing for the analysis of non-linear relationships. However, it is limited by the assumption of the existence of a breakpoint in glaucoma progression, which is still a topic of debate.^1 25^ On the other hand, the anti-log model assumes a linear relationship between variables and is more straightforward to interpret. Notably, the floor effect, a common issue in VF and RNFL data, can be equivalently measured as the intercept in the anti-log model. Both the breakpoint and anti-log models exhibit improved structure–function correlations and lower floor effects when using compensated data, highlighting the importance of accounting for compensation methods in these analyses.

### Comparison with existing methods

Several strategies have been proposed to address individual ocular factors in interpreting the relationship between structural and functional measures in patients with glaucoma. The compensation model incorporated various demographic variables to establish a multivariate normative value, with the compensated RNFL outperforming the measured RNFL. Montesano *et al* suggested a proportional change based on a normative value derived from a regression model,^26^ prompting potential future studies comparing three approaches—proportionate change, compensation and direct measurement. Recent methods involving normalisation/rotation aim to align the centres of the ONH and fovea for a more accurate VF test comparison, as described by Jansonius *et al*^6^ and Hood *et al*.^27^ This involves applying mathematical transformations to VF data based on ONH and foveal locations. Denniss *et al* developed a simulation model to explore the relationship between ONH sectors and VF locations across various anatomical parameters.^28^ While direct comparisons with these methods are challenging due to differences in study design and implementation, this study introduces a novel and comprehensive approach to compensating RNFL thickness measurements in glaucoma. It enhances our understanding of the structure–function relationship and provides a refined assessment of glaucomatous damage.

### Study strengths and limitations

The study’s strengths include a sizeable sample of 600 glaucoma-affected eyes, enhancing generalisability, and including various glaucoma stages for a comprehensive assessment of compensated RNFL thickness. Diverse structure–functional mapping techniques, such as sectoral and focal correlations, provided a thorough evaluation of the impact of compensation on the structure–function relationship in glaucoma.

However, limitations exist. The study focused on a single ethnicity, limiting generalisability to other ethnicities with potential variations in anatomical factors and RNFL thickness. Additionally, the study included subjects with low myopia levels, and findings may not fully apply to high myopes, warranting further investigation. The compensation model was solely ONH based due to the absence of macular images, potentially limiting the model’s completeness. Incorporating fovea parameters could enhance the structure–function relationship model in future research.

## Conclusions

The compensated RNFL thickness approach proves advantageous across different glaucoma stages. First, it boosts sensitivity in detecting glaucomatous damage, especially in early stages where subtle structural changes may be overlooked without compensating for anatomical variations. Second, the enhanced structure–function relationship supports monitoring disease progression in advanced stages, making it versatile for early detection and ongoing assessment. In conclusion, this study highlights that implementing compensated RNFL thickness improves the structure–function relationship in glaucoma compared with relying solely on default measured RNFL thickness values from OCT.

## supplementary material

10.1136/bjo-2023-324792 online supplemental file 1

## Data Availability

Data are available upon reasonable request.
