# Supplementary material for: Enhancing the structure–function relationship in glaucoma using anatomical compensation of retinal nerve fibre layer
Source: Br J Ophthalmol. 2024 May 7;108(12):e324792. doi: 10.1136/bjo-2023-324792 (PMC11671891; doi:10.1136/bjo-2023-324792)
Supplement: online supplemental file 1 [file bjo-108-12-s001.pdf]

## Supplementary material

**Supplementary Table 1. Demographic and ocular characteristics of glaucoma patients**

| **Characteristics** | **Glaucoma participants** |
|---|---|
| Number of participants | 412 |
| Age, years | 67.2 ± 8.9 |
| Gender, male (%) | 260 (63) |
| Number of eyes | 600 |
| Global VF MD, dB | -6.53 ± 5.55 dB; +2.03 (minimum) to -31.78 (maximum) dB |
| Glaucoma severity, n (%) | |
| Mild | 353 (59) |
| Moderate | 156 (26) |
| Advanced | 91 (15) |
| Spherical equivalent, dioptres | -1.31 ± 2.20 |
| Signal strength | 7.02 ± 0.92 |
| Global RNFL thickness, µm | |
| Measured RNFL | 75.49 ± 12.52 |
| Multivariable normative RNFL | 96.51 ± 0.41 |
| Deviation from normality after compensation approach (measured RNFL *minus* multivariable normative RNFL) | -21.02 ± 12.58 |

Values are expressed as mean ±SD unless otherwise indicated.

RNFL = retinal nerve fibre layer; VF MD = visual field mean deviation

**Supplementary Table 2.** Spearman correlations between VF mean deviation and RNFL thickness for the Garway-Heath map

| | Measured RNFL | Compensated RNFL |
|---|---|---|
| **All glaucoma** | | |
| MD (logarithm) | 0.33 | 0. 46 |
| MD (antilogarithm) | 0.30 | 0.43 |
| **Mild glaucoma** | | |
| MD (logarithm) | 0.13 | 0.21 |
| MD (antilogarithm) | 0.13 | 0.22 |
| **Moderate glaucoma** | | |
| MD (logarithm) | 0.08 | 0.19 |
| MD (antilogarithm) | 0.09 | 0.16 |
| **Advanced glaucoma** | | |
| MD (logarithm) | 0.07 | 0.18 |
| MD (antilogarithm) | 0.06 | 0.20 |

**RNFL thickness compensation model**

Vessel density profiles were extracted from OCT scans using proprietary MATLAB software, analyzing scanning laser ophthalmoscopy fundus images centered on the optic disc.[1] The software determined the thickness and position of circumpapillary retinal vessels. Integrating individual vessel positions and corresponding thickness values generated a comprehensive circumpapillary retinal vessel density profile (**Supplementary Figure 1**).

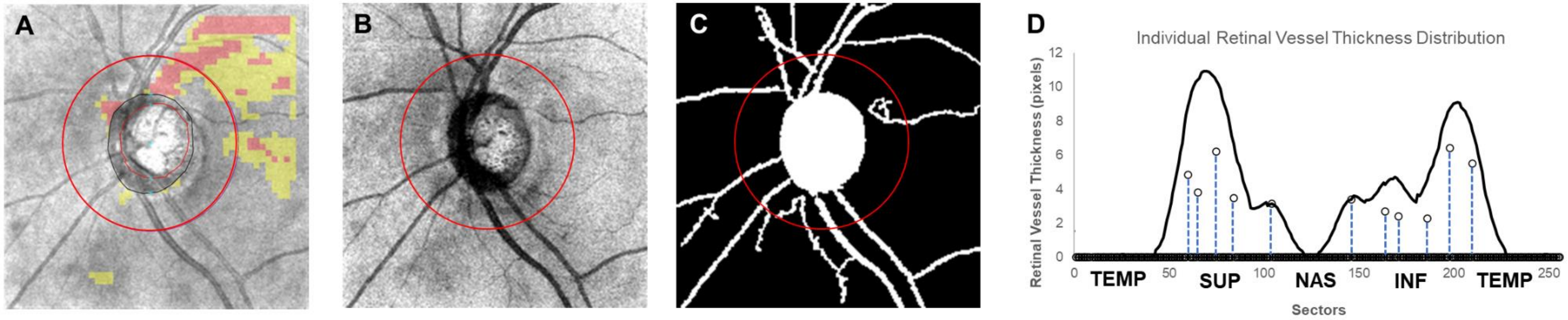


RNFL deviation map

Scanning laser ophthalmoscopy (SLO) fundus image

Binarize the SLO image to show the retinal vessel density profile.

Circumpapillary distribution of individual retinal vessel thickness values (dashed blue lines) and the representative result of the convolution with a Gaussian-shaped function (solid black line).

**Supplementary Figure 1.** Generation of the circumpapillary retinal vessel density profile.

The optic disc parameters (**Supplementary Figure 2**) are as follows: 1) area (size of the optic disc area and is measured in $mm^2$), 2) orientation (angle between the horizontal axis and the major axis of the optic disc; refers to the tilt of the optic disc and is measured in degrees), and 3) ratio (quotient between major and minor axis; refers to the shape of the optic disc, whether it is elliptical or more rounded where the ratio is closer to 1, it indicates that the major and minor axes are more similar in length, suggesting a more rounded

or circular shape of the optic disc). These variables are known to correlate with peripapillary RNFL distribution.[2-5] The coefficients of the regression equation were determined using a development dataset of healthy individuals. The model was then used to predict the multivariable normative RNFL thickness, which represents the expected RNFL thickness for a healthy participant with specific values for the compensation variables. The predicted normative values were then used as a reference to compare the RNFL thickness measurements obtained from glaucoma patients.

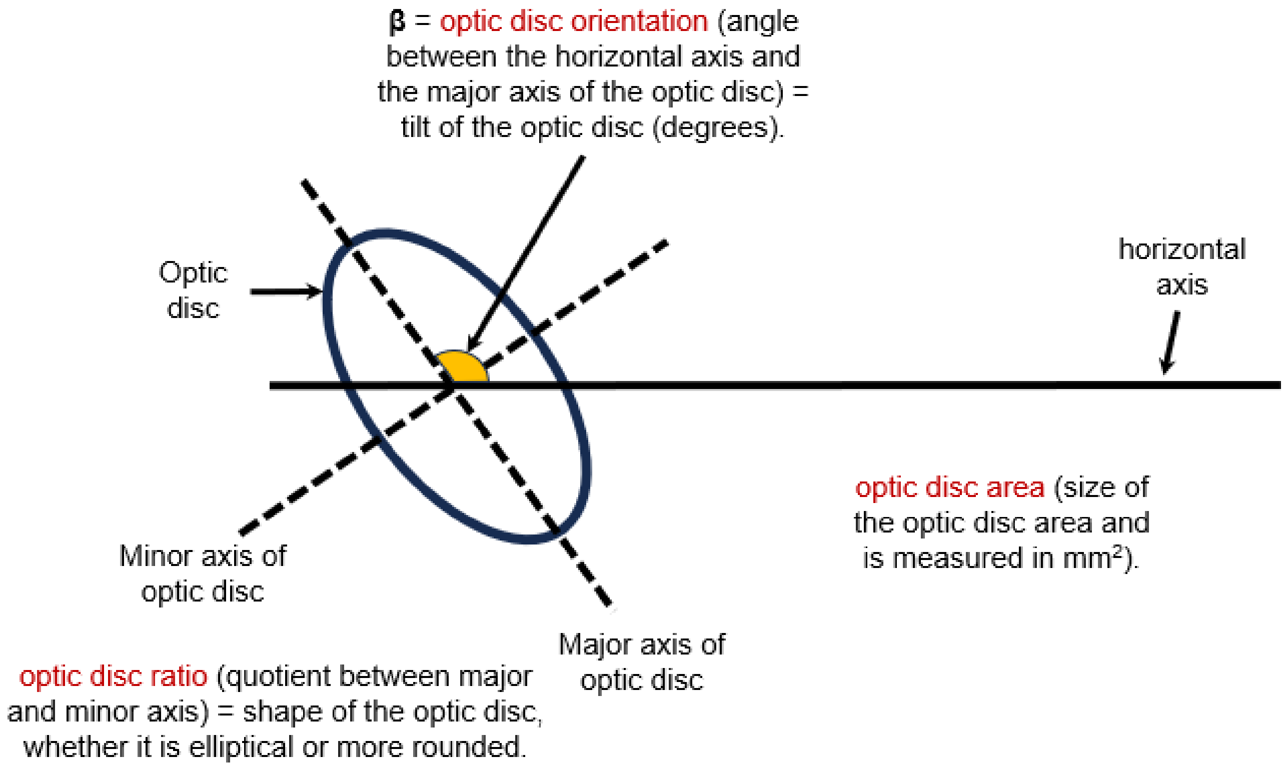


**Supplementary Figure 2.** Schematic representation of the optic disc parameters employed in creating the multivariate model. The continuous line denotes the horizontal axis. passing through the optic disc center. The dotted lines represent the major and minor axes of the optic disc. Angle "β" refers to the optic disc orientation.

The compensation model in our study generates an anatomically compensated normative profile, allowing for the calculation of deviations from normality. While the sector average thickness remains constant, the normative classification adjusts during the compensation process. Deviations are color-coded as "within normal limits" in green, borderline in yellow, and outside normal limits in red.

Additionally, we introduced an extra step in the calculation process to explore the structure-function relationship between traditional and compensation approaches. This step involves adding compensated RNFL values to the mean RNFL value from the normative database, obtained by calculating the average RNFL thickness of the healthy population.[2] The adjustment formula is as follows: compensated RNFL = (measured RNFL − multivariable normative RNFL) + mean RNFL from the normative database. This ensures meaningful visual comparisons between compensated and measured RNFL values, providing insights into the structure-function relationship.

## References

1. Pereira I, Weber S, Holzer S, et al. Correlation between retinal vessel density profile and circumpapillary RNFL thickness measured with Fourier-domain optical coherence tomography. Br J Ophthalmol 2014;**98**(4):538-43 doi: 10.1136/bjophthalmol-2013-303910[published Online First: Epub Date]|.
2. Chua J, Schwarzhans F, Nguyen DQ, et al. Compensation of retinal nerve fibre layer thickness as assessed using optical coherence tomography based on anatomical confounders. Br J Ophthalmol 2020;**104**(2):282-90 doi: 10.1136/bjophthalmol-2019-314086[published Online First: Epub Date]|.
3. Celebi AR, Mirza GE. Age-related change in retinal nerve fiber layer thickness measured with spectral domain optical coherence tomography. Invest Ophthalmol Vis Sci 2013;**54**(13):8095-103 doi: 10.1167/iovs.13-12634[published Online First: Epub Date]|.
4. Budenz DL, Anderson DR, Varma R, et al. Determinants of normal retinal nerve fiber layer thickness measured by Stratus OCT. Ophthalmology 2007;**114**(6):1046-52 doi: 10.1016/j.ophtha.2006.08.046[published Online First: Epub Date]|.
5. Hood DC, Fortune B, Arthur SN, et al. Blood vessel contributions to retinal nerve fiber layer thickness profiles measured with optical coherence tomography. J Glaucoma 2008;**17**(7):519-28 doi: 10.1097/IJG.0b013e3181629a02[published Online First: Epub Date]|.
